# Early endovascular salvage of acute inferior vena cava obstruction after liver transplantation

**DOI:** 10.1016/j.jvscit.2026.102537

**Published:** 2026-09-29

**Authors:** Emily Swafford, Grant Woodruff, Punit Vyas, Charles Zhang, Daniel Clair, Patrick Stone

**Affiliations:** Department of Vascular Surgery, Vanderbilt University Medical Center, Nashville, TN

**Keywords:** Venography, Intravascular ultrasound, Thrombectomy, Endovascular repair

## Abstract

Inferior vena cava obstruction following liver transplantation is rare but life-threatening. Although endovascular management has been described in delayed presentations, evidence supporting intervention in the immediate postoperative period remains limited. We report the case of a 43-year-old man with acute caval thrombosis and venous outflow obstruction following bicaval liver transplantation. Impending graft failure and hemodynamic instability prompted urgent endovascular intervention. Percutaneous mechanical thrombectomy was performed. Two torsion-related stenotic segments persisted, and a self-expanding bare-metal venous stent was deployed, restoring caval flow. Early thrombectomy and stenting may offer effective salvage in select high-risk patients with acute caval obstruction.

Orthotopic liver transplantation (OLT) is a lifesaving, yet technically complex, surgical procedure. Inferior vena cava (IVC) complications after liver transplantation are quite rare, occurring in less than 5% of patients; however, they carry devastating consequences with significant morbidity and mortality.^1^ Postoperative caval obstruction is one such IVC complication that arises from technical injury, torsion, or extrinsic compression, leading to impaired venous outflow and possible graft failure.^2^, ^3^

Management strategies for venous outflow obstruction from IVC thrombosis ranges from clinical observation to retransplantation.^1^ Nonetheless, guidance on surgical management remains limited. Although endovascular interventions have been reported in delayed presentations, evidence supporting their use in the immediate postoperative period is sparse.^4^, ^5^ We report the case of acute postoperative IVC thrombosis successfully managed with early mechanical thrombectomy and stenting. Written informed consent was obtained from the patient for publication of this case.

## Case presentation

A 43-year-old man with alcoholic cirrhosis presented for liver transplantation with a preoperative Model for End-Stage Liver Disease score of 22. His cirrhosis was complicated by refractory ascites with previous spontaneous bacterial peritonitis, esophageal varices requiring band ligation, hepatic encephalopathy, and portal vein thrombosis requiring chronic warfarin therapy. During transplantation, clamping of the suprahepatic vena cava resulted in caval injury with massive hemorrhage and profound hemodynamic instability. Hemostasis was ultimately achieved, and the bicaval and hepatic arterial anastomoses were completed. The blood loss during the operation was estimated to be 20 L, and the patient required massive transfusion with 29 units of packed red blood cells, 44 units of fresh frozen plasma, and additional platelets, cryoprecipitate, and fluid. Despite hemorrhage control and completion of the vascular anastomoses, the patient remained profoundly hemodynamically unstable, requiring 30 μg/min of norepinephrine and 0.06 U/min of vasopressin. Given his persistent instability, severe coagulopathy, and ongoing resuscitation requirements, temporary abdominal closure was performed, and the patient was taken to the surgical intensive care unit. By postoperative day 2, the patient’s hemodynamics had improved, and he returned to the operating room for biliary reconstruction and abdominal closure. At that time, intraoperative findings included large volume hematoma and IVC thrombus. Open thrombectomy via cavotomy was performed with copious clot removal and excellent forward bleeding. Despite mechanical thrombectomy by the liver transplant surgery team, postoperative laboratory values suggested impending graft failure, and computed tomography imaging demonstrated intrahepatic transplant vena cava thrombus and concern for impaired venous outflow (Fig 1).

**Fig 1:**
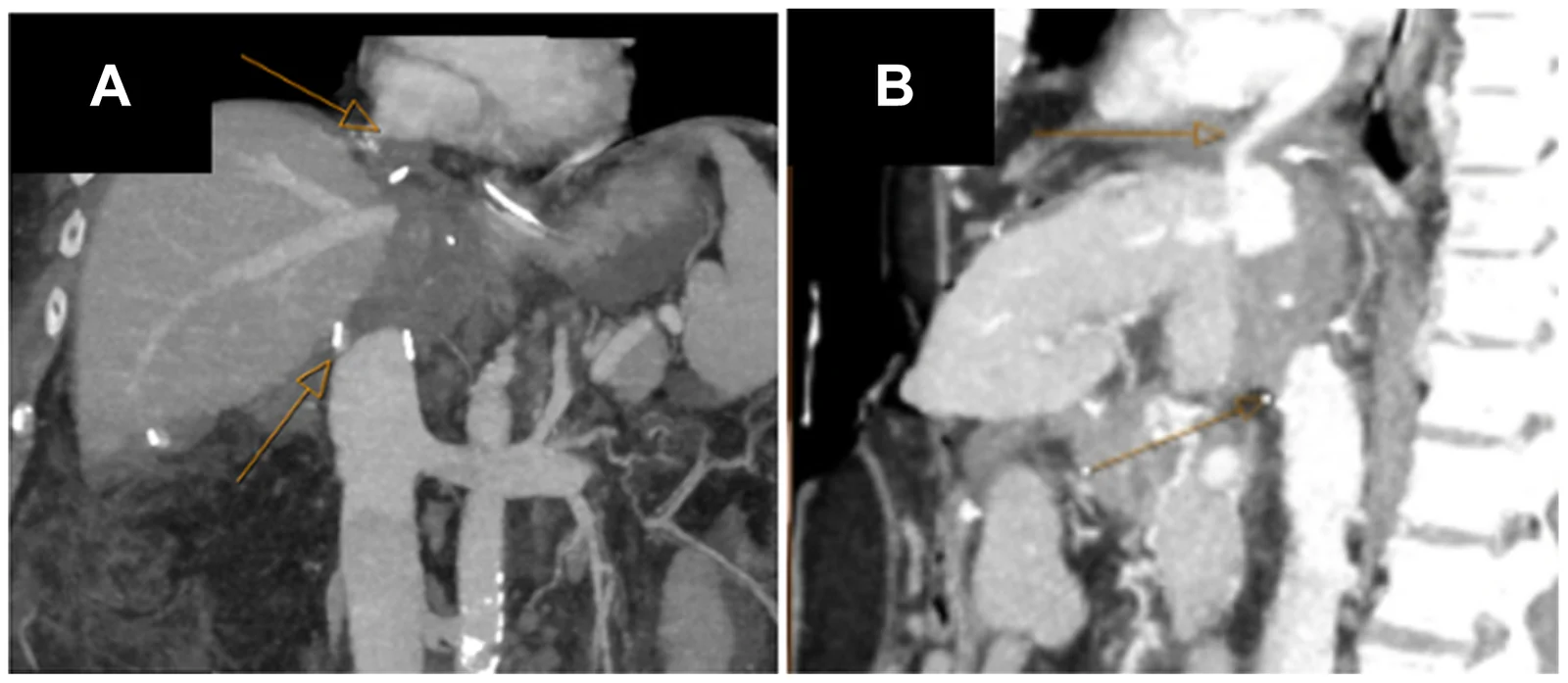
Preoperative imaging with acute inferior vena cava occlusion. Computed tomography demonstrating intrahepatic transplant caval thrombus near its insertion into the native inferior vena cava in the coronal **(A)** and sagittal **(B)** views.

Given the patient’s hemodynamic instability, there was concern that the patient would not survive repeat open surgical revision. Therefore, a multidisciplinary decision was made to pursue endovascular intervention with the vascular surgery team. The patient underwent urgent venography with intravascular ultrasound guidance. Following femoral vein access, venocavography demonstrated markedly diminished caval flow. Inferior traversal of the IVC was achieved; however, superior access was not possible because of a left-sided superior vena cava (SVC), precluding distal embolic protection (Fig 2). Given the severity of the obstruction and clinical necessity for flow restoration, percutaneous mechanical thrombectomy was performed with a 16F Penumbra Lightning Flash catheter (Penumbra, Inc.) without the use of a distal embolic protective device.

**Fig 2:**
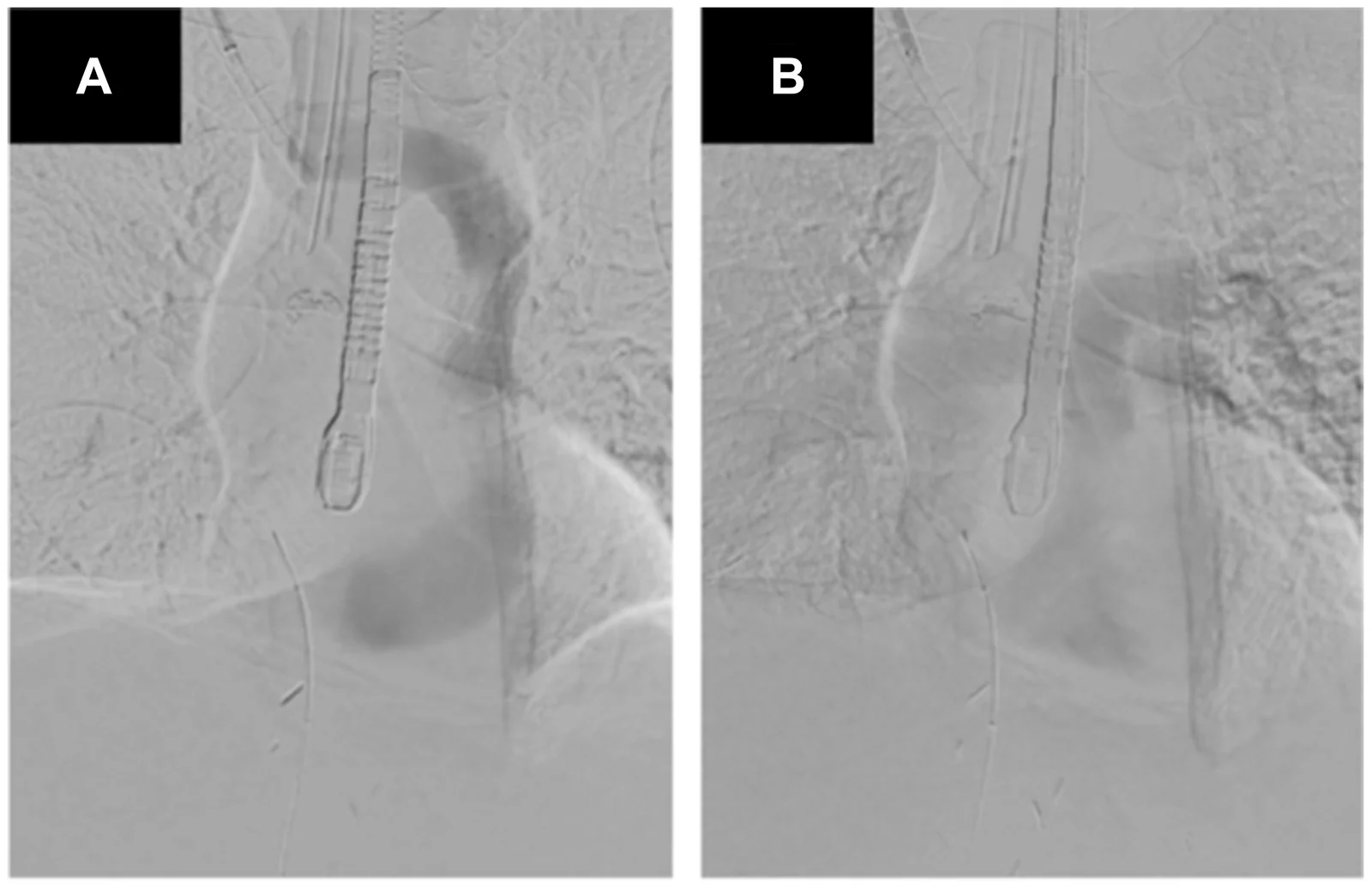
Left-sided superior vena cava. Left-sided superior vena cava entering coronary sinus and right heart **(A)** followed by the right ventricle, right ventricular outflow tract, and right and left pulmonary arteries **(B)**.

Post-thrombectomy imaging revealed two residual stenotic segments likely related to caval torsion. The proximal segment was at the suprahepatic IVC near its entry into the right atrium, and the distal segment was at the level of the infrahepatic caval anastomosis (Fig 3, *A*). Balloon angioplasty failed to adequately resolve the recalcitrant lesions. Therefore, a 20 by 120 mm, 9F, Abre, self-expanding, bare-metal venous stent was deployed from the right atrium to the suprarenal IVC. Subsequent postdilation with 16 and 20 mm angioplasty balloons resulted in significant luminal gain, and completion imaging confirmed restoration of unobstructed caval flow (Fig 3, *B*). In addition, intraoperative transesophageal echocardiogram revealed improved right ventricular filling.

**Fig 3:**
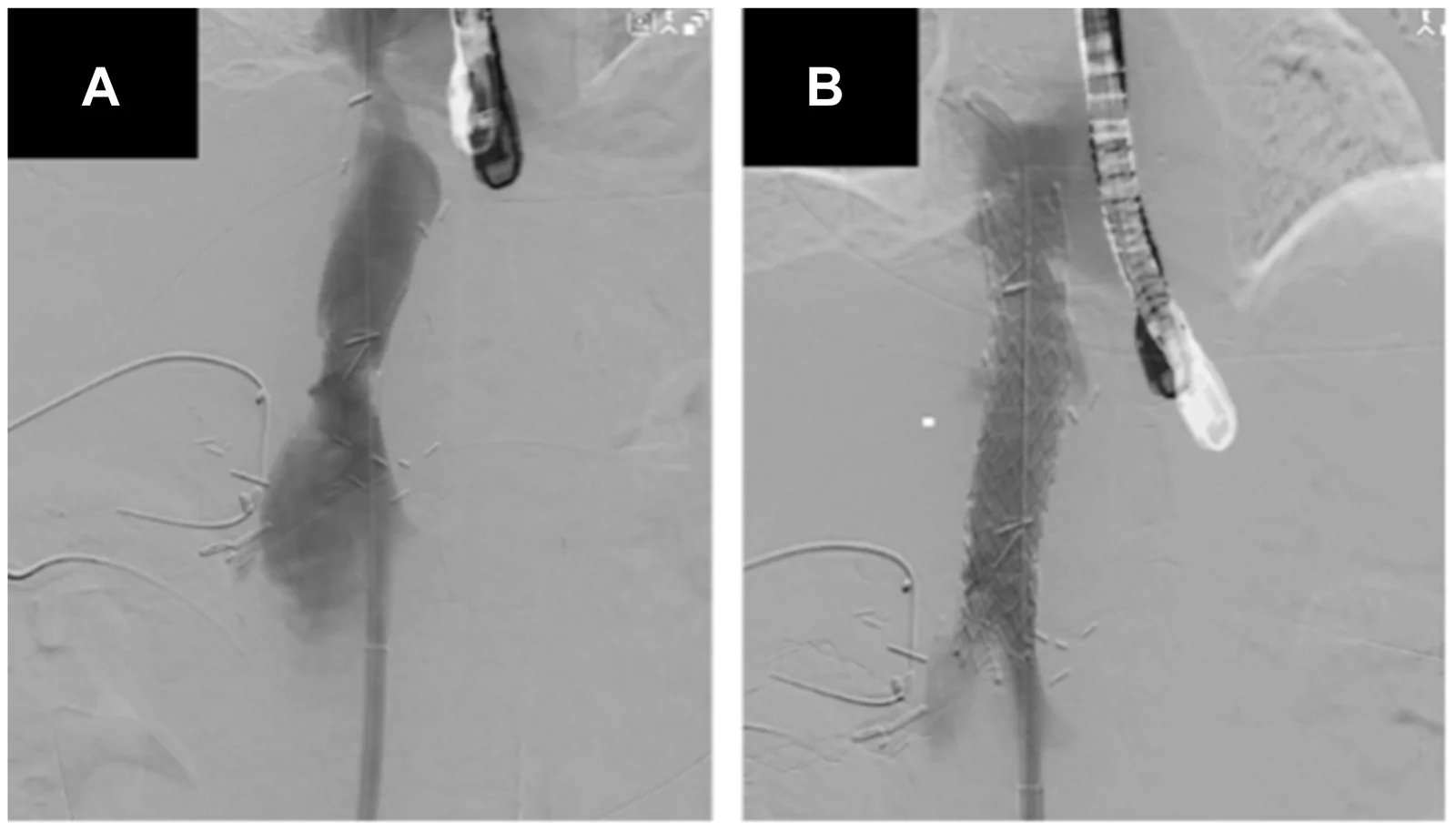
Venocavography before and after endovascular stenting. **A,** Venocavography following mechanical aspiration thrombectomy demonstrating severe residual stenosis of the suprahepatic and infrahepatic vena cava. **B,** Completion venocavography after deployment of a self-expanding bare-metal venous stent.

The patient’s postoperative course was unfortunately complicated by a cerebrovascular event resulting in transient right-sided hemiplegia and dysphagia. He ultimately recovered and stepped down from the intensive care unit 2 weeks after his index operation. He was discharged home on hospital day 20 on apixaban 5 mg twice daily and aspirin 81 mg daily. Before discharge, imaging confirmed IVC patency (Fig 4). At 1-year follow-up, he has remained clinically stable without symptoms of recurrent IVC occlusion, and surveillance imaging demonstrated continued stent patency (Fig 5).

**Fig 4:**
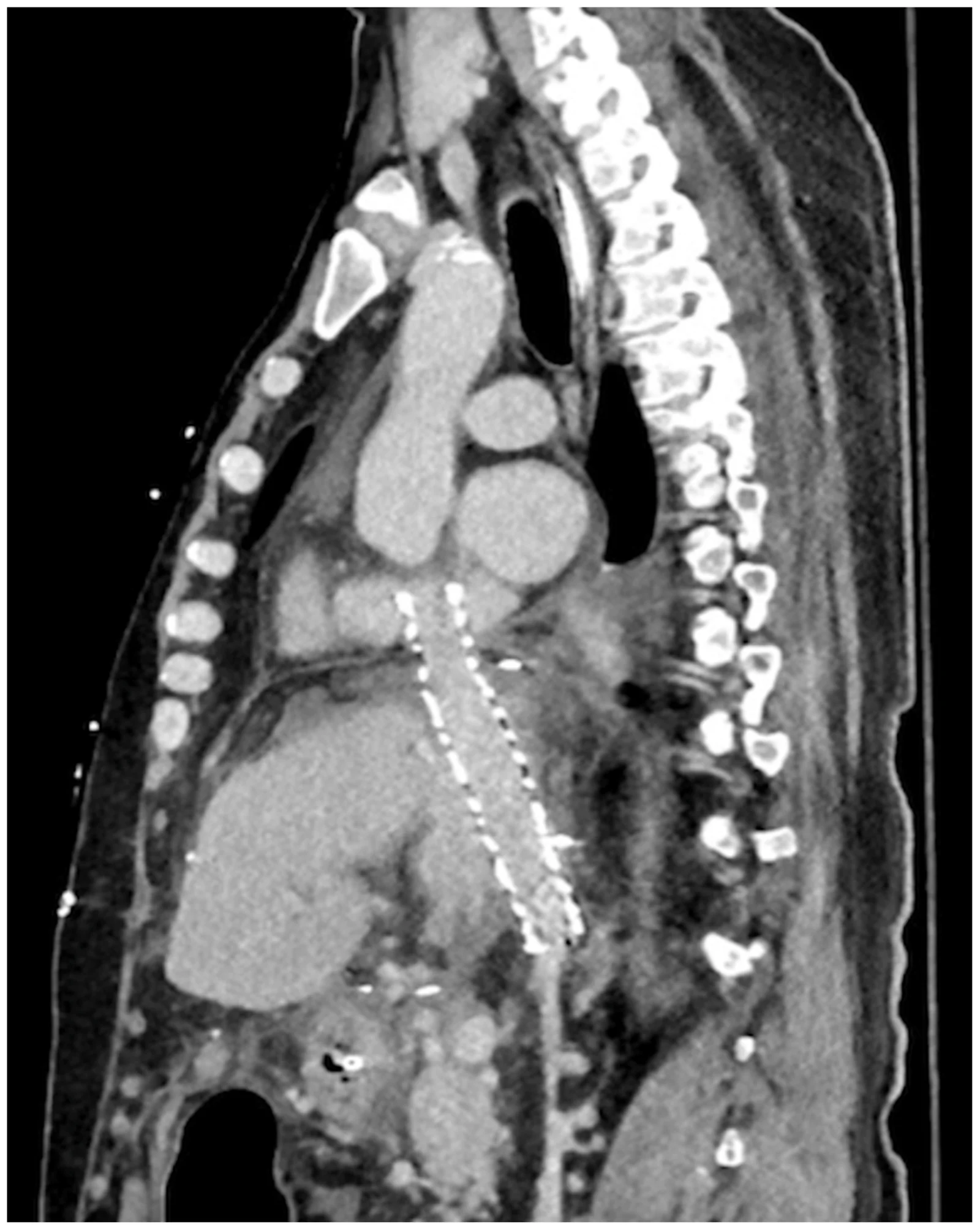
Postoperative imaging demonstrating caval stent patency. Sagittal computed tomography demonstrating patency of the inferior vena cava stent following mechanical thrombectomy and endovascular stent placement.

**Fig 5:**
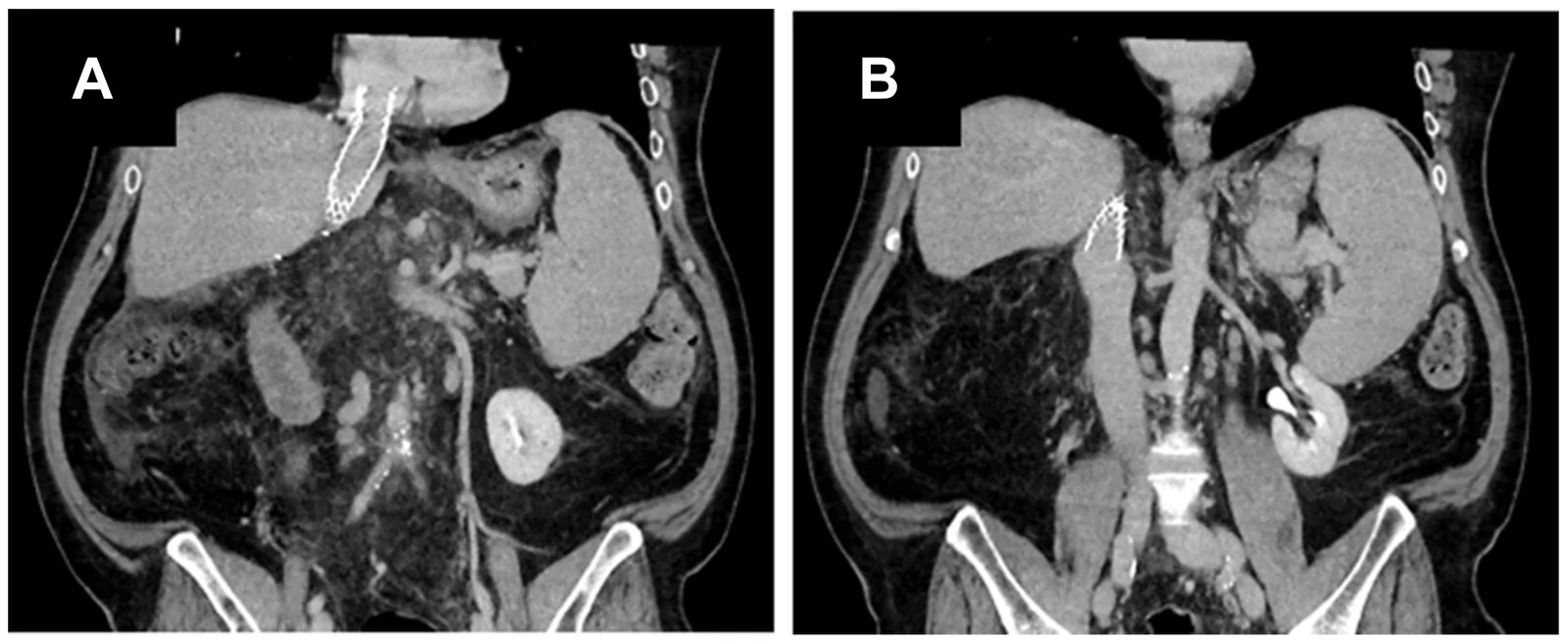
Longitudinal imaging demonstrating sustained stent patency. Axial computed tomography demonstrating continued patency of the inferior vena cava stent at 1 year postoperatively at the superior **(A)** and inferior **(B)** portions of the stent.

## Discussion

Acute IVC obstruction following OLT is a graft- and life-threatening complication with limited guidance regarding optimal management. Existing literature describes delayed presentations of venous outflow obstruction treated with endovascular approaches.^6^ However, these cases are several weeks postoperative and not applicable for immediate postoperative caval thrombosis. Early intervention is still traditionally managed with open surgical revision in the immediate postoperative period.^7^, ^8^ In patients with hemodynamic instability and prohibitive risk for open surgical revision, however, the current guidelines fall short. This case aids in closing the critical knowledge gap that exists for procedural planning and decision-making when managing acute caval obstruction in the immediate postoperative period.

Anastomotic technique in liver transplantation should also be considered when contemplating solutions for caval complications. Options include conventional end-to-end cavocavostomy with bicaval anastomosis, side-to-side cavocaval anastomosis, and piggyback anastomosis with the donor suprahepatic IVC anastomosed to a common cloaca of the recipient hepatic veins. Although bicaval, side-to-side, and piggyback techniques have not demonstrated significant differences in the rates of outflow obstruction, anatomical factors may impact anastomotic torsion and determine types of intervention possible if outflow obstruction were to develop.^9^ In this case, bicaval reconstruction was performed because of a large liver size and caudate lobe anatomy. However, given the technical challenges and intraoperative hemorrhage encountered in this case, the caval reconstruction may have resulted in caval torsion, contributing to subsequent caval thrombosis. Although endovascular thrombectomy and stenting successfully restored caval patency, it did not correct the underlying anatomic distortion. Persistent caval torsion may predispose to recurrent outflow obstruction, stent thrombosis or deformation, and hepatic venous congestion, underscoring the importance of longitudinal surveillance for recurrent caval stenosis and graft dysfunction.^10^

This case was further complicated by an isolated, persistent left SVC with an absent right SVC, which is seen in 0.09% to 0.13% of the general population.^11^ Persistent left SVC drains via the coronary sinus then into the right atrium, limiting access to the IVC via right internal jugular vein. The inability to obtain proximal control precluded the use of an embolic protection device during our mechanical thrombectomy. Although the overall risk of stroke following IVC thrombectomy is low, the presence of anomalous venous anatomy may increase the risk of paradoxical embolization and could have contributed to this patient’s postoperative cerebrovascular insult.^12^, ^13^

Our work demonstrates that in carefully selected patients, early endovascular therapy with thrombectomy and stenting can be performed successfully and may provide a viable alternative to high-risk, open intervention. This report highlights the critical role of venography and intravascular ultrasound for diagnosis and guiding treatment of acute caval complications. It also underscores the importance of early vascular surgery involvement and multidisciplinary collaboration in managing complex transplant patients. Further investigation is needed to define the safety, durability, and long-term outcomes of early endovascular intervention in this setting.

## Conclusions

Early mechanical thrombectomy and IVC stenting can serve as a safe and effective salvage strategy for acute postoperative caval obstruction following OLT. Prompt endovascular intervention, guided by a multidisciplinary approach, can restore venous outflow and preserve graft function in these high-risk transplant patients.

## Funding

None.

## Disclosures

None.
